# Trends in Retinopathy of Prematurity and Strabismus Among Preterm and Low-Birth-Weight Infants in Korea: A Nationwide Population-Based Study

**DOI:** 10.3390/children13101424

**Published:** 2026-10-17

**Authors:** Jaehyeong Cho, Hyeonji Shin, Jun Chul Byun, Ji Hye Jang

**Affiliations:** 1 Department of Research, Keimyung University Dongsan Medical Center, Daegu 42601, Republic of Korea; jaehyeongcho@dsmc.or.kr; 2 Department of Statistics, Kyungpook National University, Daegu 41566, Republic of Korea; guswl0507@knu.ac.kr; 3 Department of Pediatrics, Keimyung University School of Medicine, Daegu 42601, Republic of Korea; jcbyun@dsmc.or.kr; 4 Department of Ophthalmology, Keimyung University School of Medicine, Daegu 42601, Republic of Korea

**Keywords:** retinopathy of prematurity, strabismus, infant, premature, low birth weight

## Abstract

**Highlights:**

**What are the main findings?**

- From 2010 to 2022, the claims-recorded incidence of retinopathy of prematurity (ROP) and strabismus among preterm and low-birth-weight infants in Korea declined significantly, despite increasing proportions of infants born at earlier gestational ages and with lower birth weights.
- Gestational age and birth weight were associated with recorded diagnoses for both ROP and strabismus, with the highest incidence of both conditions observed among the most immature infants, particularly those born at <28 weeks of gestation and weighing < 1000 g.

**What are the implications of the main findings?**

- Despite the decline in recorded diagnoses in ROP and strabismus, risk-based ophthalmic screening and follow-up should remain intensive for infants born at earlier gestational ages and lower birth weights.
- The steeper decline in strabismus than in ROP among recent birth cohorts may partly reflect shorter follow-up, underscoring the need for long-term ophthalmic surveillance to detect strabismus beyond the neonatal period.

**Abstract:**

Background/Objectives: In Korea, the proportion of preterm and low-birth-weight infants has risen even as total births have fallen, changing the population at risk for eye disease of prematurity. Whether nationwide trends in retinopathy of prematurity (ROP) and strabismus reflect true temporal change or the shifting composition of this high-risk birth cohort is unclear. We examined 13-year trends in both conditions and assessed whether they persisted after adjustment for major risk factors. Methods: We analyzed nationwide Health Insurance Review and Assessment Service claims from 2010 to 2022. Infants born at a gestational age below 37 weeks and/or with a birth weight below 2500 g were included. ROP and strabismus were identified from diagnostic codes, and gestational age and birth weight strata from perinatal diagnosis codes. Multivariable logistic regression estimated adjusted odds ratios for calendar-year trends and for joint gestational age and birth weight subgroups. Results: Among 195,551 eligible infants, ROP was recorded in 55,457 (28.4%) and strabismus in 48,301 (24.7%). Claims-recorded cumulative incidence of both conditions declined from 2010 to 2022 even though the cohort became progressively more immature. After adjustment, the odds in 2022 relative to 2010 were 0.27 (95% confidence interval, 0.25–0.28) for ROP and 0.17 (0.16–0.18) for strabismus. Risk remained concentrated in the most immature infants: among those born below 28 weeks with a birth weight below 1000 g, the adjusted odds ratio was 21.54 (19.41–23.91) for ROP and 2.74 (2.52–2.97) for strabismus. The decline was not uniform. Among infants born below 28 weeks, ROP stayed between 73% and 84% throughout with no downward trend, whereas strabismus fell from 55.9% to 15.7%. Conclusions: Claims-recorded ROP and strabismus declined nationwide from 2010 to 2022 despite an increasingly vulnerable birth cohort, and these declines persisted after adjustment. Gestational age and birth weight remained the dominant determinants of risk, supporting continued risk-based screening. These findings do not establish trends in severe ROP, and the apparent decline in strabismus should be interpreted cautiously because later birth cohorts had shorter follow-up.

## 1. Introduction

Retinopathy of prematurity (ROP) and strabismus are two of the most common ophthalmic complications of preterm birth, and the risk of each rises as gestational age (GA) and birth weight (BW) fall [1,2,3]. ROP begins with arrest of retinal vascularization, followed by abnormal neovascularization that can progress to retinal detachment; it remains a major cause of childhood blindness [4,5,6]. Strabismus impairs binocular vision, predisposes to amblyopia, and can have lasting functional and psychosocial effects [7,8]. Because preterm and low-birth-weight (LBW) infants undergo routine ROP screening and subsequent ophthalmic follow-up, the recorded incidence of these conditions reflects the quality of neonatal and ophthalmic care as well as biological risk.

The epidemiologic context of preterm birth in Korea has changed substantially. The country has one of the lowest birth rates in the world, while advancing maternal age, wider use of assisted reproductive technology, and improved survival of extremely preterm infants have increased the number of surviving preterm and LBW infants [9,10,11,12,13]. The birth cohort has therefore become progressively more vulnerable even as births fell, and on demographic grounds alone the incidence of ROP and strabismus would be expected to rise. At the same time, improvements in neonatal intensive care, oxygen management, and standardized ROP screening and treatment have reduced the risk of severe ROP and other vision-threatening complications [13,14,15]. Two opposing forces have thus operated simultaneously.

Earlier Korean studies reported a declining ROP incidence but focused on ROP alone and did not account for the changing risk profile of the birth cohort [16,17]. National trends in strabismus in this population have not been comprehensively evaluated. We therefore used 13 years of nationwide claims data to characterize both trends, to test whether they persisted after adjustment for GA, BW, sex, and insurance type, and to quantify risk across GA and BW subgroups.

## 2. Materials and Methods

### 2.1. Data Source and Study Population

We conducted a nationwide, population-based cohort study using claims from the Health Insurance Review and Assessment Service (HIRA), the single agency that reviews reimbursement claims under Korea’s mandatory National Health Insurance. Because enrollment is compulsory, HIRA records virtually all encounters nationwide, with diagnoses coded by the Korean Standard Classification of Diseases (KCD), an adaptation of the International Classification of Diseases, 10th Revision. This coverage suits long-term trend analysis [18,19]. Claims were extracted for January 2009 to December 2023. Infants born in 2009 or 2023 were not eligible: perinatal records for 2009 may be truncated because the database begins that January, and the 2023 cohort had no subsequent year for outcomes to accrue, which matters particularly for strabismus. The cohort therefore comprised infants born in 2010 to 2022 who were preterm (GA < 37 weeks) and/or had LBW (BW < 2500 g), identified from perinatal diagnosis codes. The infant was the unit of analysis, with records de-duplicated on the encrypted beneficiary identifier. Infants with discrepant multiple GA or BW codes were excluded because the stratum could not be assigned unambiguously. Infants recorded as dying in hospital within the first year were also excluded, so that competing mortality did not differentially reduce the opportunity for outcomes to be recorded across birth years. Only in-hospital deaths are ascertainable from claims, so this removes the identifiable component of competing mortality rather than all deaths.

### 2.2. Outcome and Covariate Definition

ROP and strabismus were identified by at least one claim with the relevant diagnostic codes in any position, principal or secondary. ROP was defined by H35.1 codes and strabismus by H49 (paralytic strabismus) and H50 (other strabismus) codes. Repeat claims or specialist confirmation were not required. This claim-based ROP definition does not capture disease stage as defined by ICROP3 classification [20] and follows previous Korean claim-based studies [16,17]. Prematurity and LBW were identified from codes P07.0 (<1000 g), P07.1 (1000 to 2499 g), P07.2 (GA < 28 completed weeks), and P07.3 (GA 28 to <37 completed weeks). Infants were categorized by GA (<28 or 28 to <37 weeks) and by BW (<1000 or 1000 to 2499 g). Because P07 codes identify only preterm birth or low birth weight, infants without a code on one axis were assigned to the corresponding reference category (GA ≥ 37 weeks or BW ≥ 2500 g). These reference categories do not represent verified term or normal-birth-weight infants. Covariates were birth year, sex from the beneficiary registry, and insurance type (National Health Insurance or Medical Aid), with Medical Aid used as a proxy for low household socioeconomic status. Each infant was counted once per each outcome, and infants with both conditions were included in both analyses.

### 2.3. Statistical Analysis

For each birth-year cohort, cumulative incidence was the number of infants with a recorded diagnosis through 2023 divided by the at-risk infants born that year, with each outcome assigned to the birth year regardless of age at diagnosis. Follow-up duration varied across birth cohorts; these proportions do not represent cumulative incidence over a common follow-up period. Incidence was calculated overall and within GA and BW strata. Two multivariable logistic regression models were fitted. Model 1 estimated adjusted odds ratios (aORs) with 95% confidence intervals (CIs) for calendar year, GA, BW, sex, and insurance type, with 2010 as reference. Model 2 replaced the GA and BW terms with a combined GA-by-BW variable, using GA 28 to <37 weeks with BW ≥ 2500 g as reference. In a sensitivity analysis, the stratified trend description was repeated using BW rather than GA as the stratifying variable. No adjustment was made for multiple comparisons, because the calendar-year estimates were interpreted collectively as describing the shape of the trend rather than as independent tests. Analyses used R version 4.4.1 (R Foundation for Statistical Computing, Vienna, Austria). All tests were two-sided, and *p*-values < 0.05 were considered significant.

## 3. Results

### 3.1. Cohort Characteristics

Of 208,358 preterm and/or LBW infants born in 2010 to 2022, 8026 (3.9%) were excluded for discrepant multiple perinatal codes and 4781 for in-hospital death within the first year, leaving 195,551. During the entire follow-up period, ROP was recorded at least once in 55,457 infants (28.4%) and strabismus in 48,301 infants (24.7%), representing the overall cumulative incidence of each condition. Because follow-up duration varied by birth year, the opportunity for these diagnoses to be recorded also differed across birth cohorts. Of the total cohort, 92,207 infants (47.2%) were female and 10,877 (5.6%) were Medical Aid beneficiaries. By GA, 4138 (2.1%) were coded at <28 weeks and 166,999 (85.4%) at 28 to <37 weeks; 24,414 (12.5%) carried no GA code and were classified as ≥37 weeks. By BW, 5659 (2.9%) were coded at <1000 g and 122,091 (62.4%) at 1000 to 2499 g; 67,801 (34.7%) carried no BW code and were classified as ≥2500 g (Table 1).

Cumulative incidence rose with greater immaturity, more steeply for ROP: 78.1% at GA < 28 weeks versus 7.6% in the reference group, and 75.3% at BW < 1000 g versus 20.0% in the reference group. The corresponding strabismus figures were 43.6% versus 20.7% and 45.2% versus 23.5%.

### 3.2. Trends in Cumulative Incidence

**Figure 1:**
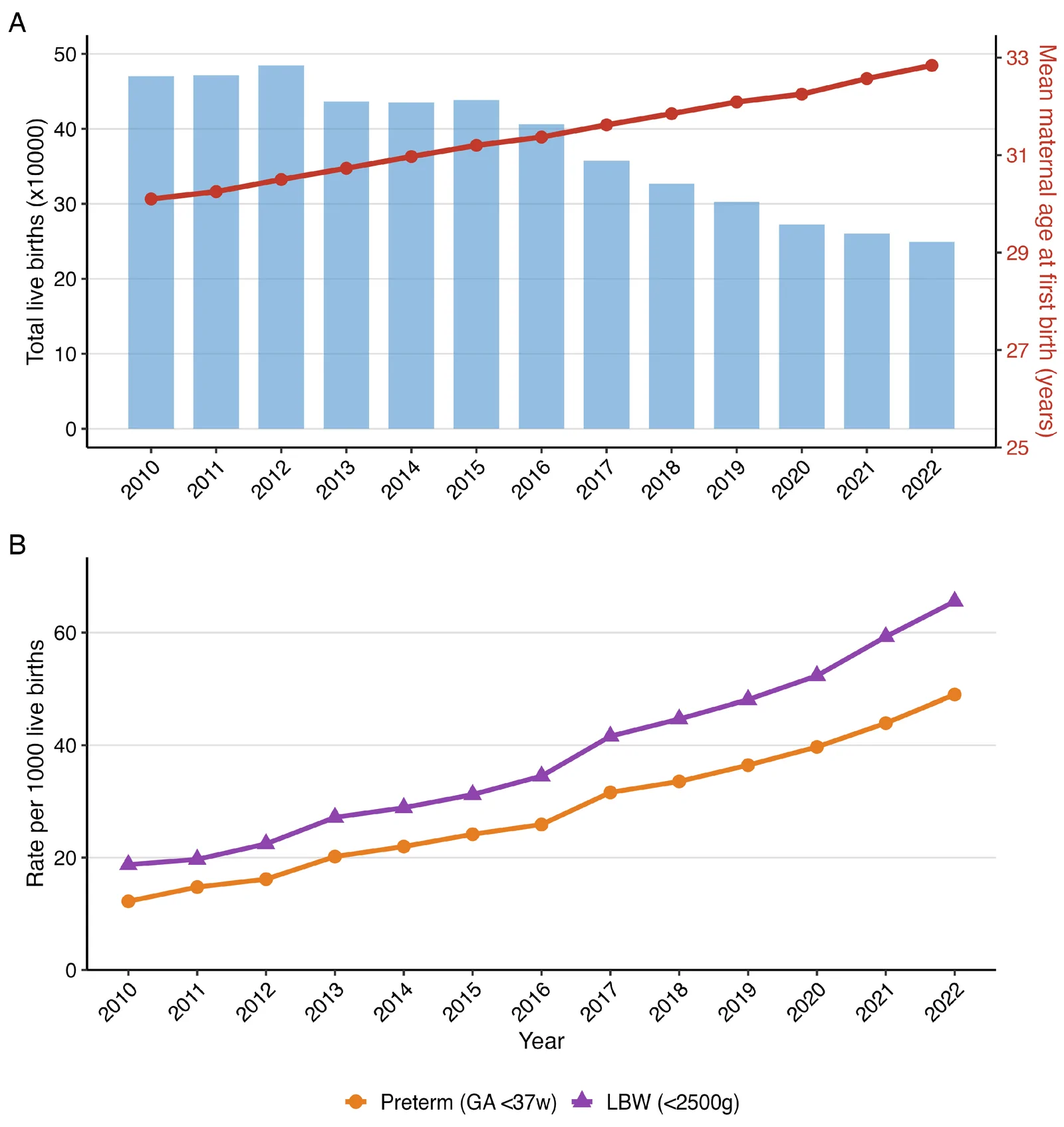
National demographic context of live births in Korea from 2010 to 2022. (**A**) Annual number of total live births (bars; left y-axis; units of 10,000 births) and mean maternal age at first birth (line; right y-axis; years). (**B**) Claims-recorded rates of preterm birth (gestational age < 37 weeks) and low birth weight (<2500 g) per 1000 live births. The annual number of live births declined by approximately 47% during the study period, whereas the claims-recorded rates of preterm birth and low birth weight increased. These trends likely reflect a combination of epidemiologic changes—including increasing maternal age and the use of assisted reproductive technology—and improved survival and diagnostic ascertainment of high-risk infants.

Total live births fell from 470,171 in 2010 to 249,186 in 2022, mean maternal age at first birth rose from 30.1 to 32.8 years, and preterm and LBW infants recorded per 1000 live births rose over the same period. The relative burden of preterm and LBW infants therefore increased as the birth cohort contracted (Figure 1).

**Figure 2:**
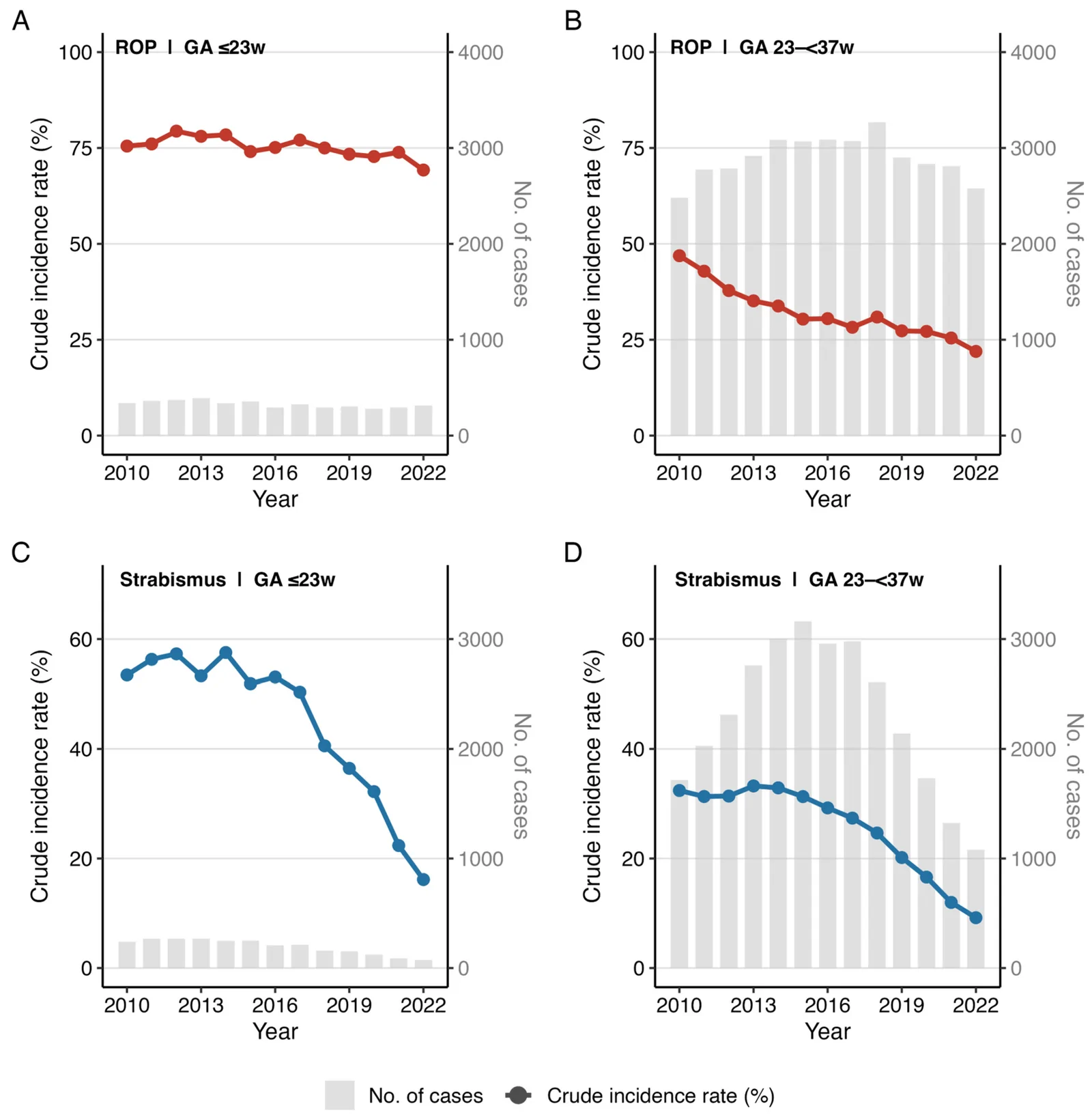
Annual number of cases and crude cumulative incidence of retinopathy of prematurity and strabismus by gestational age group, 2010 to 2022. (**A**) Retinopathy of prematurity among infants with a gestational age < 28 weeks. (**B**) Retinopathy of prematurity among infants with a gestational age of 28 to <37 weeks. (**C**) Strabismus among infants with a gestational age < 28 weeks. (**D**) Strabismus among infants with a gestational age of 28 to <37 weeks. Gray bars show the annual number of cases (right ordinate); the line with circles shows the crude cumulative incidence (left ordinate, %). The incidence ordinate is shared within each disease row (0 to 100% for retinopathy of prematurity, 0 to 70% for strabismus) to allow direct comparison between gestational age strata. Denominators are the annual number of infants in each stratum, defined by Korean Standard Classification of Diseases codes P07.2 and P07.3. Diagnoses were counted through December 2023, with shorter follow-up for later birth cohorts.

**Figure A1:**
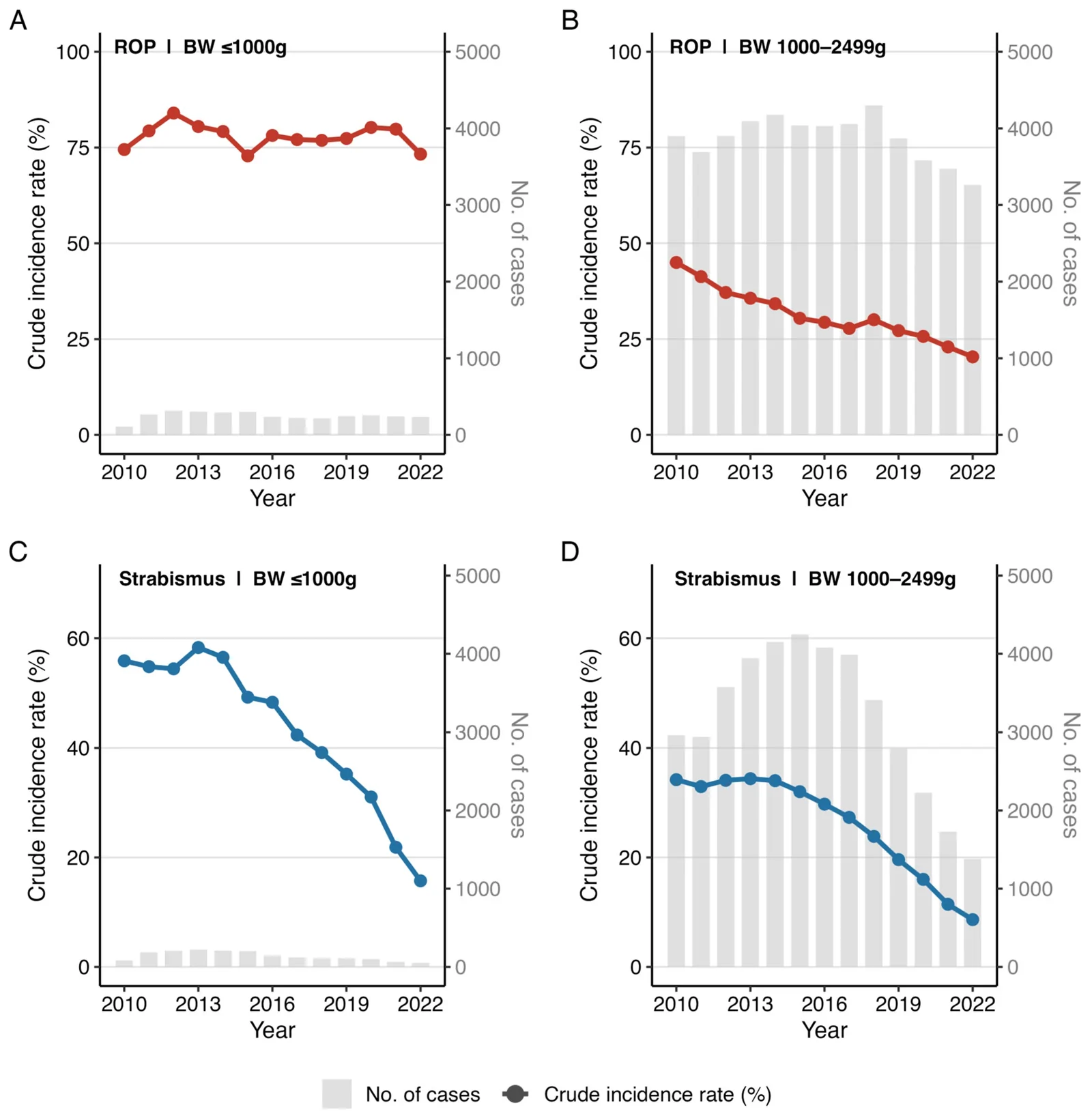
Annual number of cases and crude cumulative incidence of retinopathy of prematurity and strabismus by birth weight group, 2010 to 2022. (**A**) Retinopathy of prematurity among infants with a birth weight < 1000 g. (**B**) Retinopathy of prematurity among infants with a birth weight of 1000 to 2499 g. (**C**) Strabismus among infants with a birth weight < 1000 g. (**D**) Strabismus among infants with a birth weight of 1000 to 2499 g. Gray bars show the annual number of cases (right ordinate); the line with circles shows the crude cumulative incidence (left ordinate, %). The incidence ordinate is shared within each disease row (0 to 100% for retinopathy of prematurity, 0 to 70% for strabismus). Diagnoses were counted through December 2023, with shorter follow-up for later birth cohorts.

Cumulative incidence nevertheless fell in most strata, though not uniformly. At GA 28 to <37 weeks, ROP fell from 45.0% to 20.4% and strabismus from 34.2% to 8.6%. At GA < 28 weeks, strabismus fell markedly, from 55.9% to 15.7%, whereas ROP showed no downward trend, ranging from 73% to 84% across the period (74.5% in 2010, 73.3% in 2022) (Figure 2). The BW-stratified analysis showed a similar pattern (Figure A1).

**Figure 3:**
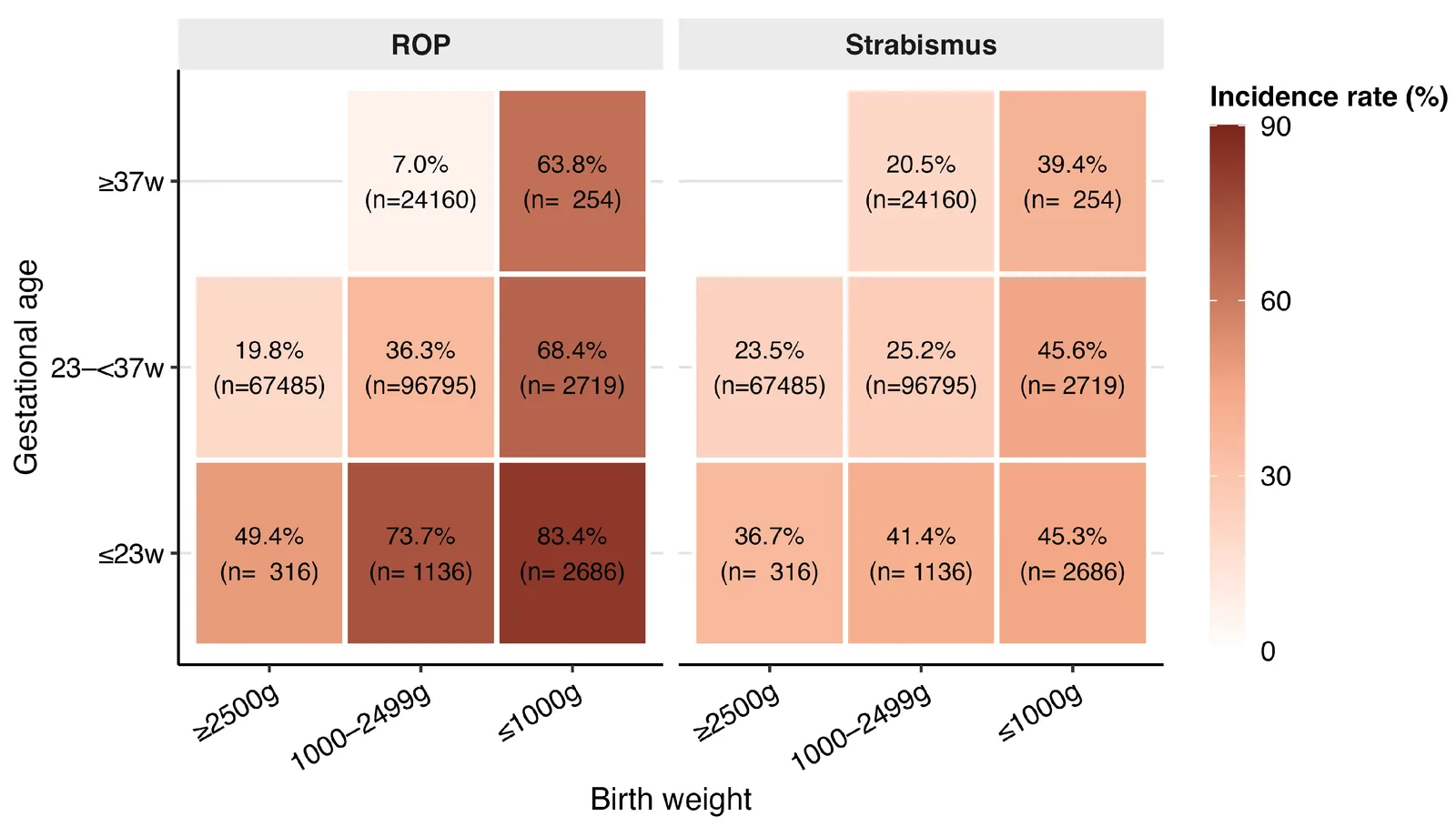
Crude cumulative incidence of retinopathy of prematurity and strabismus across joint gestational age and birth weight strata, 2010 to 2022. Each cell shows the crude cumulative incidence (%) with the number of infants in the stratum. Color intensity is proportional to the incidence. The cell for gestational age ≥ 37 weeks with birth weight ≥ 2500 g is not shown because infants with neither a prematurity nor a low-birth-weight code were not eligible for the cohort. The GA ≥ 37-week and BW ≥ 2500 g labels denote categories assigned when the corresponding code was absent and do not establish actual GA or BW. Diagnoses were counted through December 2023, with follow-up duration varying by birth year.

Risk increased with immaturity across joint strata. In the most immature subgroup (GA < 28 weeks with BW < 1000 g), ROP incidence was 83.4% versus 7.0% among infants classified as ≥37 weeks with BW 1000 to 2499 g, an approximately 12-fold difference; the corresponding strabismus figures were 45.3% and 20.5%, an approximately 2-fold difference. The immaturity-related gradient was therefore substantially steeper for ROP (Figure 3).

### 3.3. Multivariable Analysis

In Model 1, GA and BW were the strongest risk factors for both outcomes, with substantially larger effects for ROP (Table 2). Compared with the reference group, GA < 28 weeks was associated with an aOR of 24.31 (95% CI, 22.05–26.80) for ROP and 1.89 (95% CI, 1.73–2.05) for strabismus. Compared with BW ≥ 2500 g, BW < 1000 g was associated with aORs of 8.63 (95% CI, 8.02–9.29) for ROP and 2.27 (95% CI, 2.12–2.43) for strabismus. Female sex was associated with slightly lower odds of ROP (aOR, 0.90; 95% CI, 0.88–0.92) and no change for strabismus (aOR, 1.00; 95% CI, 0.98–1.03). Medical Aid coverage carried lower recorded odds of both outcomes, particularly strabismus (aOR, 0.49; 95% CI, 0.46–0.51).

**Figure 4:**
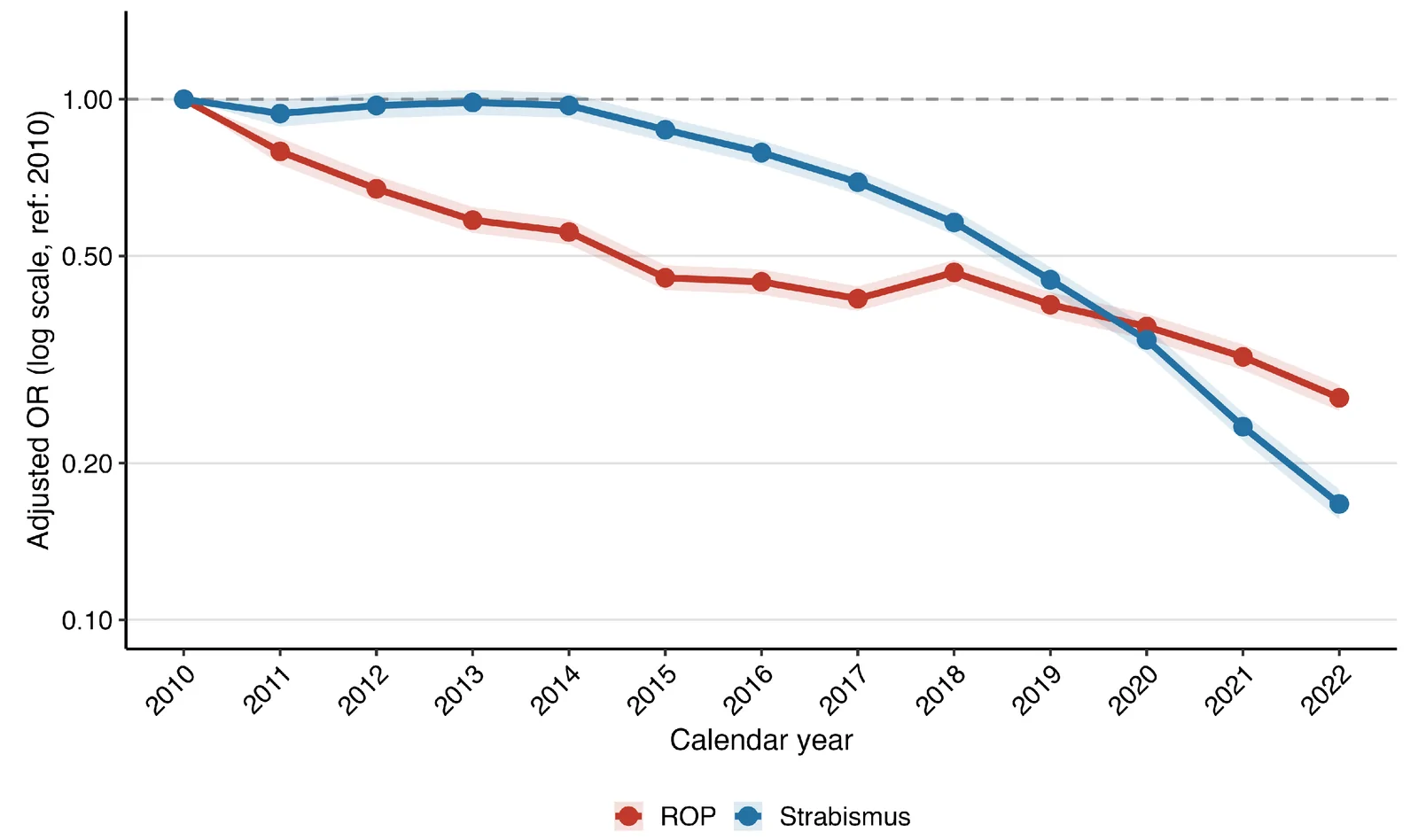
Adjusted calendar-year odds ratios for retinopathy of prematurity and strabismus, 2011 to 2022, with 2010 as the reference. Lines are adjusted odds ratios from Model 1 and shaded bands are 95% confidence intervals, on a logarithmic scale. The horizontal dashed line indicates the reference odds ratio of 1.0. Estimates are adjusted for gestational age, birth weight, sex, and insurance type. Circles denote retinopathy of prematurity and triangles denote strabismus. Numeric estimates are given in Table A1.

Calendar year remained independently associated with lower odds of both conditions after adjustment (Table A1). Relative to 2010, the 2022 aOR was 0.27 (95% CI, 0.25–0.28) for ROP and 0.17 (95% CI, 0.16–0.18) for strabismus, reductions of approximately 73% and 83%. The trajectories differed: the ROP odds ratio fell steadily from the start of the study period, whereas the strabismus odds ratio remained close to unity until approximately 2015 and declined thereafter (Figure 4).

In Model 2, risk rose as GA and BW fell within the preterm strata, relative to the reference subgroup (GA 28 to <37 weeks with BW ≥ 2500 g) and was highest at GA < 28 weeks with BW < 1000 g (Table 3), where the aOR was 21.54 (95% CI, 19.41–23.91) for ROP and 2.74 (95% CI, 2.52–2.97) for strabismus. BW remained informative within the ≥37 weeks group. Infants with BW < 1000 g had increased odds of ROP (aOR, 6.45; 95% CI, 4.96–8.37), although this stratum is small (*n* = 254) with internally discordant codes, so the estimate should be read with caution. Those with BW 1000 to 2499 g had lower odds than the reference (aOR, 0.31; 95% CI, 0.29–0.33).

## 4. Discussion

Using 13 years of nationwide claims data, we found that the claims-recorded cumulative incidence of ROP and strabismus in Korea declined across the 2010–2022 birth cohorts, including after adjustment for GA, BW, sex, and insurance type. However, ROP remained frequent among infants born at <28 weeks and did not show a clear downward trend in this group. The recorded patterns differed: among infants born below 28 weeks, strabismus fell steeply while ROP did not decline at all.

These findings are consistent with previous Korean evidence: survival of very LBW infants improved, particularly at the lowest GAs [12,13], and Hong et al. [16] reported a fall in ROP incidence from 39.5% in 2007 to 23.5% in 2018. Our study extends this work through the 2022 birth cohort, shows that the ROP decline persisted after adjustment for the changing composition of the at-risk population, and demonstrates a parallel decline in strabismus, not previously characterized in this population. Similar ROP declines despite improved survival of very preterm infants have been reported elsewhere, although trends have not been uniform [6,21,22].

Changes in neonatal care, including oxygen management, respiratory support, infection control, antenatal corticosteroid use and surfactant, may have contributed to changes in ROP risk [12,13,15]. Advances in screening, classification, and treatment may also have influenced the detection and clinical outcomes of ROP [14,20,23,24,25,26]. However, the present claims data cannot determine the contribution of any specific interventions to the observed trend.

The non-uniformity of the decline is the finding of greatest clinical relevance. Two explanations, not mutually exclusive, are plausible. The first is a ceiling effect: when most of a stratum is affected, there is little room for further reduction. The second, and in our view more important, is a shift in case mix within the stratum. Because survival at the lowest gestational ages improved over the same period [12], a growing proportion of infants coded below 28 weeks were probably born at the limits of viability and survived to undergo screening. The exclusion of in-hospital deaths compounds this: as survival improved, those who would previously have died before screening increasingly entered the analyzed cohort. Better care and greater immaturity within one code category act in opposite directions and may cancel each other out. Our data cannot separate these influences, because the four-character P07.2 category spans all GAs below 28 weeks without gradation. The Korean Neonatal Network holds week-level data but enrolls only very low-birth-weight infants and does not capture strabismus, so comparing both conditions in one national cohort required claims data.

The practical consequence is the same whatever the mechanism. Recorded ROP declined mainly among moderately preterm infants, but these data cannot establish whether severe or treatment-requiring ROP changed in either group. Screening capacity should therefore not be reallocated away from this group on the strength of the favorable overall trend [14].

The association between immaturity and risk was considerably stronger for ROP, which is biologically plausible. ROP arises directly from arrested retinal vascular development and aberrant neovascularization, processes tightly coupled to prematurity and oxygen exposure. Strabismus reflects a broader spectrum of neurodevelopmental, sensory, and motor pathway abnormalities, to which prematurity contributes but does not dominate, and it occurs at appreciable frequency in the general pediatric population [27]. Previous cohorts have likewise found GA and BW to be stronger predictors of ROP than of strabismus [1,2,28]. In practice, GA and BW serve well as a triage rule for ROP but discriminate far less well for strabismus, which argues against follow-up strategies that are simply scaled versions of the ROP screening criteria. Because earlier detection widens the range of non-surgical management options, follow-up extending into the preschool years is warranted [29].

The magnitude of the strabismus decline warrants caution, being larger than changes in neonatal care alone would predict. The trajectory is informative: the strabismus odds ratio remained close to unity for the first half of the period and then fell steeply, whereas the ROP odds ratio declined from the start. Much childhood strabismus is diagnosed in the preschool years [30], so infants born later had less opportunity to accrue a diagnosis before the observation window closed. Differential follow-up may contribute to this pattern, although its contribution cannot be determined from the present analyses. A fixed follow-up or time-to-event analysis would be required to more clearly distinguish genuine temporal changes from differential censoring. Because such analyses were not performed in the present study, the apparent decline in strabismus, particularly in the more recent birth cohorts, should be interpreted cautiously.

A second caution concerns the denominator. The observed increase in claims-recorded preterm and LBW infants per 1000 live births may reflect a combination of true demographic and epidemiologic changes, improved neonatal survival, and more complete use of P07 diagnostic codes over time. If infants at progressively lower risk entered the denominator, part of the decline would follow arithmetically from dilution rather than improved outcomes. The decline persisted after adjustment for GA and BW and was present within individual strata, which argues against dilution operating only between strata. Composition within strata may still have shifted, because each spans a wide range of GA and BW, and this cannot be excluded with code-based categories.

The lower recorded odds of both conditions among Medical Aid beneficiaries deserve comment. Medical Aid covers low-income households, and the association differed between outcomes: the deficit was marked for strabismus but slight for ROP. ROP is detected during protocol-driven inpatient screening, largely independent of family circumstances, whereas strabismus is identified at outpatient follow-up requiring repeated caregiver-initiated visits over several years. Differential ascertainment is a possible explanation, but sustained follow-up after discharge was not directly evaluated. The lower claims-recorded diagnostic rates in this group may reflect reduced access to healthcare, differences in healthcare utilization, and consequent under-ascertainment or coding differences rather than a true reduction in biological risk. The finding should not be interpreted as a protective biological effect.

The strengths are the nationwide design, which minimizes selection bias and yields generalizable estimates; the 13-year window, which permits assessment of trends rather than a single cross-section; and identical case definitions within one cohort, allowing direct comparison of the two trajectories. Several limitations should also be considered. First, both conditions were identified from codes rather than clinical examination, so disease stage could not be assessed; stage-specific ROP codes entered the KCD only in 2021. Therefore, the observed downward trend reflects a decline in the claims-recorded incidence of any ROP but does not necessarily indicate a corresponding reduction in severe, treatment-requiring, or sight-threatening ROP. Because severity determines the clinical significance of ROP, this is the most consequential gap. Second, the H49 and H50 codes encompass heterogeneous strabismus subtypes, not all specifically related to prematurity, and subtype-level analysis was not possible. Cases were also defined from a single recorded code without a requirement for repeat visits or specialist attribution, which may admit some misclassification. Furthermore, defining each outcome based on a single occurrence of the relevant diagnostic code may have made the incidence estimates susceptible to changes in coding practices or diagnostic criteria over the 13-year study period, potentially influencing the observed temporal trends. Accordingly, incomplete or differential ascertainment across birth cohorts and insurance groups cannot be excluded. Third, GA and BW were derived from perinatal codes rather than record review, and covariates including oxygen-targeting practice, sepsis, bronchopulmonary dysplasia, and maternal conditions could not be assessed [31]. The characteristics of the 8026 infants excluded because of discrepant GA or BW codes could not be evaluated, and potential selection bias resulting from their exclusion cannot be excluded. The cohort is also smaller than the national population of extremely immature infants, reflecting code-based ascertainment and the exclusion of in-hospital deaths, which disproportionately removes the least mature infants. In addition, the GA ≥ 37-week and BW ≥2500 g reference categories included infants without a corresponding P07 code and should not be interpreted as verified term or normal-birth-weight groups. Fourth, the follow-up available to each birth cohort differed by design; the consequences are discussed above. Finally, the analysis is descriptive: the adjusted calendar-year estimates quantify how much of the change is not attributable to measured covariates, not its cause.

In summary, claims-recorded diagnoses of ROP and strabismus in Korea declined across the 2010–2022 birth cohort, but these findings should not be interpreted as verified reductions in clinical disease. ROP remained common among the least mature infants, supporting continued screening intensity in this group. For strabismus, sustained ophthalmic follow-up beyond the neonatal period remains important, and the apparent decline should be interpreted cautiously because recent birth cohorts had shorter follow-up.

## Figures and Tables

**Table 1:** Baseline characteristics and crude incidence of retinopathy of prematurity and strabismus.

Characteristic	Infants	Retinopathy of Prematurity	Strabismus
Gestational age			
<28 weeks	4138 (2.1)	3232 (78.1)	1803 (43.6)
28 to <37 weeks	166,999 (85.4)	50,380 (30.2)	41,435 (24.8)
≥37 weeks	24,414 (12.5)	1845 (7.6)	5063 (20.7)
Birth weight			
<1000 g	5659 (2.9)	4261 (75.3)	2556 (45.2)
1000 to 2499 g	122,091 (62.4)	37,667 (30.9)	29,798 (24.4)
≥2500 g	67,801 (34.7)	13,529 (20.0)	15,947 (23.5)
Sex			
Male	103,344 (52.8)	30,155 (29.2)	25,489 (24.7)
Female	92,207 (47.2)	25,302 (27.4)	22,812 (24.7)
Insurance type			
National Health Insurance	184,674 (94.4)	52,124 (28.2)	46,573 (25.2)
Medical Aid	10,877 (5.6)	3333 (30.6)	1728 (15.9)
Total	195,551 (100.0)	55,457 (28.4)	48,301 (24.7)

Values are presented as number (%). Percentages in the Infants column represent the total cohort, whereas those in the outcome columns represent within-stratum crude cumulative incidence. Infants born at ≥37 weeks were included based on low birth weight, and those weighing ≥ 2500 g were included based on prematurity. The GA ≥ 37-week and BW ≥ 2500 g labels denote the reference categories assigned when the corresponding code was absent, rather than verified GA or BW values. Observation ended in December 2023 and follow-up duration varied by birth year.

**Table 2:** Adjusted odds ratios for retinopathy of prematurity and strabismus (Model 1).

Variable	Retinopathy of Prematurity	Strabismus
Gestational age		
≥37 weeks	1.00 (reference)	1.00 (reference)
28 to <37 weeks	7.36 (7.01–7.74)	1.35 (1.31–1.40)
<28 weeks	24.31 (22.05–26.80)	1.89 (1.73–2.05)
Birth weight		
≥2500 g	1.00 (reference)	1.00 (reference)
1000 to 2499 g	2.42 (2.37–2.48)	1.16 (1.13–1.18)
<1000 g	8.63 (8.02–9.29)	2.27 (2.12–2.43)
Sex		
Male	1.00 (reference)	1.00 (reference)
Female	0.90 (0.88–0.92)	1.00 (0.98–1.03)
Insurance type		
National Health Insurance	1.00 (reference)	1.00 (reference)
Medical Aid	0.93 (0.89–0.97)	0.49 (0.46–0.51)

Values are presented as adjusted odds ratio (95% confidence interval). Models were adjusted for calendar year, gestational age, birth weight, sex, and insurance type. The reference GA and BW categories include infants without the corresponding code and do not represent verified term or normal-birth-weight groups.

**Table 3:** Adjusted odds ratios for retinopathy of prematurity and strabismus across joint gestational age and birth weight strata (Model 2).

Gestational Age	Birth Weight	Retinopathy of Prematurity	Strabismus
≥37 weeks	1000 to 2499 g	0.31 (0.29–0.33)	0.85 (0.82–0.89)
≥37 weeks	<1000 g	6.45 (4.96–8.37)	1.91 (1.47–2.47)
28 to <37 weeks	≥2500 g	1.00 (reference)	1.00 (reference)
28 to <37 weeks	1000 to 2499 g	2.43 (2.37–2.48)	1.15 (1.12–1.18)
28 to <37 weeks	<1000 g	8.58 (7.88–9.33)	2.60 (2.40–2.82)
<28 weeks	≥2500 g	3.66 (2.93–4.59)	1.64 (1.30–2.08)
<28 weeks	1000 to 2499 g	11.56 (10.10–13.22)	2.22 (1.96–2.51)
<28 weeks	<1000 g	21.54 (19.41–23.91)	2.74 (2.52–2.97)

Values are presented as adjusted odds ratio (95% confidence interval). Models were adjusted for calendar year, sex, and insurance type. The ≥37 weeks and ≥2500 g stratum was not estimable because these infants were not eligible for the study cohort. GA ≥ 37 weeks and BW ≥ 2500 g denote categories assigned in the absence of the corresponding code, not verified values.

## Data Availability

The claims data analyzed in this study are held by the Health Insurance Review and Assessment Service and cannot be redistributed by the authors. Access may be requested through the HIRA healthcare big data hub.

## Abbreviations

The following abbreviations are used in this manuscript:

ROP	Retinopathy of Prematurity
HR	Hazard Ratio
OR	Odds Ratio
GA	Gestational Age
BW	Birth Weight

## Appendix A

### Appendix A.1

Values are presented as adjusted odds ratio (95% confidence interval), adjusted for gestational age, birth weight, sex, and insurance type, with 2010 as the reference year. These estimates are shown graphically in Figure 4 of the main article. Odds ratios are given to two decimal places throughout; the upper confidence limit for strabismus in 2011 is 0.996 and therefore excludes 1.00.

**Table A1:** Adjusted calendar-year odds ratios for retinopathy of prematurity and strabismus, 2011 to 2022 (Model 1).

Calendar Year	Retinopathy of Prematurity	Strabismus
2010	1.00 (reference)	1.00 (reference)
2011	0.79 (0.75–0.84)	0.94 (0.89–1.00)
2012	0.67 (0.64–0.71)	0.97 (0.92–1.03)
2013	0.59 (0.55–0.62)	0.99 (0.93–1.04)
2014	0.56 (0.53–0.59)	0.97 (0.92–1.03)
2015	0.45 (0.43–0.48)	0.87 (0.83–0.92)
2016	0.45 (0.42–0.47)	0.79 (0.75–0.83)
2017	0.41 (0.39–0.44)	0.69 (0.66–0.73)
2018	0.47 (0.44–0.49)	0.58 (0.55–0.61)
2019	0.40 (0.38–0.43)	0.45 (0.42–0.48)
2020	0.37 (0.35–0.39)	0.34 (0.32–0.37)
2021	0.32 (0.30–0.34)	0.23 (0.22–0.25)
2022	0.27 (0.25–0.28)	0.17 (0.16–0.18)
